# Supplementary material for: The Antiviral and Virucidal Activities of Voacangine and Structural Analogs Extracted from Tabernaemontana cymosa Depend on the Dengue Virus Strain
Source: Plants (Basel). 2021 Jun 23;10(7):1280. doi: 10.3390/plants10071280 (PMC8309144; doi:10.3390/plants10071280)
Supplement: Supplementary file 1 [file plants-10-01280-s001.zip › Table S1 (14-05-2021).pdf]

Table S1. ^{13}C and ^{1}H NMR nuclear magnetic resonance assignments of Voacangine-7-hydroxyindolenine (**TcK002)** isolated from the ethanol extract of seeds of *Tabernaemontana cymosa* Jacq., comparing to those reported in the literature.

	**TcK002**		**[1]**		**[2]**		**[3]**	
**Carbon**	^{13}C	^{1}H	^{13}C	^{1}H	^{13}C	^{1}H	^{13}C	^{1}H
**2**	186,97		186,8		188,1		186,8	
**3**	48,75		48,6		48,7		48,6	
**5**	49,22		49		48,9		49,1	
**6**	34,25		34,1		32,2		34,2	
**7**	88,43		88,3		87,3		88,3	
**8**	144,53		144,4		144,9		144,4	
**9**	108,09	6,91	107,9	6,9	108,4		107,9	6,94
**10**	159,23		159,1		158,4		159,1	
**11**	113,85	6,8	113,7	6,81	113,3		113,7	6,84
**12**	121,49	7,36	121,3	7,37	121,6		121,3	7,4
**13**	144,90		144,8		143,9		144,8	
**14**	27,08		27		28,4		27	
**15**	32,14		32		30,5		32	
**16**	55,88		58,5		57,5		58,5	
**17**	34,63		34,5		36,7		34,5	
**18**	11,71	0,86	11,5	0,86	11,7		11,5	0,9
**19**	26,62		26,5		27,1		26,5	
**20**	37,68		37,5		38,9		37,5	
**21**	58,68		58,4		55,5		58,5	
**22**	174,05		173,9		172,6		173,8	
**CO_2CH_3**	53,39	3,7	55,3	3,7	52,5		53,1	3,74
**OCH_3**	55,88	3,82	55,7	3,81	54,9		55,7	3,85

1. Pereira, P.S.; França, S.d.C.; Oliveira, P.V.A.d.; Breves, C.M.d.S.; Pereira, S.I.V.; Sampaio, S.V.; Nomizo, A.; Dias, D.A. Chemical constituents from Tabernaemontana catharinensis root bark: a brief NMR review of indole alkaloids and in vitro cytotoxicity. *Química Nova* **2008**, *31*, 20-24.
2. Sharma, P.; Cordell, G.A. Heyneanine hydroxyindolenine, a new indole alkaloid from Ervatamia coronaria var. plena. *Journal of natural products* **1988**, *51*, 528-531.
3. Madinaveitia Martin, A. Alcaloides de las especies Rauwolfia sprucei, Stemmadenia obovata, Delphinium cardipetalum y Withania aristata: Contribución a la química de los alcaloides tipo Iboga. **1998**.
