# Supplementary material for: The Antiviral and Virucidal Activities of Voacangine and Structural Analogs Extracted from Tabernaemontana cymosa Depend on the Dengue Virus Strain
Source: Plants (Basel). 2021 Jun 23;10(7):1280. doi: 10.3390/plants10071280 (PMC8309144; doi:10.3390/plants10071280)
Supplement: Supplementary file 1 [file plants-10-01280-s001.zip › Table S2 (14-05-2021).pdf]

Table S2. ^{13}C and ^{1}H NMR nuclear magnetic resonance assignments of Rupicoline (**TcK004)** isolated from the ethanol extract of seeds of *Tabernaemontana cymosa* Jacq., comparing to those reported in the literature.

	**TcK004**		**[1]**		**[2]**	
**Carbon**	**^{13}C**	**^{1}H**	**^{13}C**	**^{1}H**	**^{13}C**	**^{1}H**
**2**	68,39		68,5		68,5	
**3**	52,07		52,1		51,9	
**5**	47,67		47,4		47,4	
**6**	25,77		26,1		25,7	
**7**	202,90		202,9		203	
**8**	121,78		121,7		153,9	
**9**	104,63	7,02	104,5	7,02	104,5	7,06
**10**	153,82		153,6		154,1	
**11**	126,89	7,07	126,5	7,06	126,5	7,1
**12**	114,12	6,76	112,1	6,74	113,8	6,78
**13**	154,21		154		153,3	
**14**	31,10		30,7		26,1	
**15**	31,10		25,7		31,1	
**16**	52,12		51,9		52	
**17**	30,80		31,1		30,7	
**18**	12,14	0,91	12	0,9	11,9	0,94
**19**	28,69		28,6		28,6	
**20**	35,81		35,8		35,8	
**21**	52,05	3,95	51	3,9	51	
**22**	174,57		174,8		174,8	
**CO2CH3**	52,07	3,3	51,7	3,3	51,7	3,33
**OCH3**	55,90	3,76	55,7	3,76	55,7	3,8

1. Husain, K.; Said, I.M.; Din, L.B.; Takayama, H.; Kitajima, M.; Aimi, N. Alkaloids from The Roots of Tabernaemontana Macrocarpa Jack. *Natural Product Sciences* **1997**, *3*, 42-48.
2. Madinaveitia Martin, A. Alcaloides de las especies Rauwolfia sprucei, Stemmadenia obovata, Delphinium cardipetalum y Withania aristata: Contribución a la química de los alcaloides tipo Iboga. **1998**.
