# Supplementary material for: The Antiviral and Virucidal Activities of Voacangine and Structural Analogs Extracted from Tabernaemontana cymosa Depend on the Dengue Virus Strain
Source: Plants (Basel). 2021 Jun 23;10(7):1280. doi: 10.3390/plants10071280 (PMC8309144; doi:10.3390/plants10071280)
Supplement: Supplementary file 1 [file plants-10-01280-s001.zip › Table S3 (14-05-2021).pdf]

Table S3. ^{13}C and ^{1}H NMR nuclear magnetic resonance assignments of 3-Oxo-voacangine (**TcK005**) isolated from the ethanol extract of seeds of *Tabernaemontana cymosa* Jacq., comparing to those reported in the literature.

	**TcK005**		**[1]**		**[2]**	
**Carbon**	**^{13}C**	**^{1}H**	**^{13}C**	**^{1}H**	**^{13}C**	**^{1}H**
**2**	134,65		134,5		136	
**3**	173,07		172,8		176	
**5**	42,72		42,6		42,7	
**6**	21,18		21		21,2	
**7**	109,22		108,8		109,5	
**8**	128,23		127,9		-	
**9**	100,48	6,95	100,2	6,9	100,1	6,93
**10**	154,21		153,8		154,0	
**11**	112,59	6,84	112,3	6,81	112,7	6,83
**12**	111,38	7,16	111,2	7,37	111,6	7,16
**13**	130,83		130,7		130	
**14**	35,97		35,2		38,4	
**15**	31,03		30,8		31	
**16**	56,11		55,4		56,7	
**17**	35,49		35,7		36	
**18**	11,41	0,97	11,8	0,86	11,1	1
**19**	27,67		27,5		27,6	
**20**	38,21		38		35,6	
**21**	56,02		55,8		56,1	
**22**	175,60		175,6		172,9	
**CO_2CH_3**	53,09	3,77	52,8	3,7	53	3,75
**OCH_3**	55,60	3,88	55,9	3,81	56,1	3,88

1. Sharma, P.; Cordell, G.A. Heyneanine hydroxyindolenine, a new indole alkaloid from Ervatamia coronaria var. plena. *Journal of natural products* **1988**, *51*, 528-531.
2. Goncalves, M.d.S.; Braz-Filho, R.; Mathias, L.; Vieira, I.J.C. Chemical constituents of Tabernaemontana catharinensis (apocynaceae).
