# Supplementary figures and images for: The Antiviral and Virucidal Activities of Voacangine and Structural Analogs Extracted from Tabernaemontana cymosa Depend on the Dengue Virus Strain
Source: Plants (Basel). 2021 Jun 23;10(7):1280. doi: 10.3390/plants10071280 (PMC8309144; doi:10.3390/plants10071280)

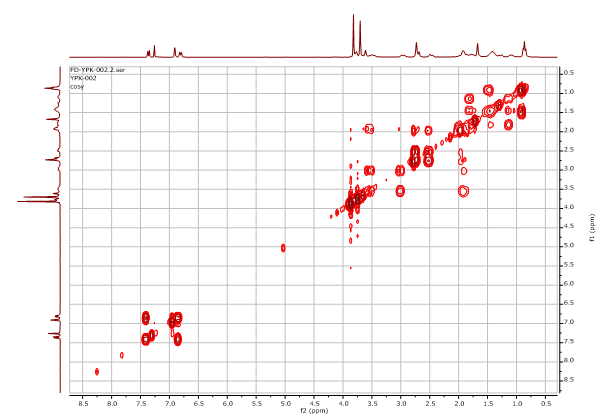

Supplement: Supplementary file 1 [file plants-10-01280-s001.zip › Figure S1 (14-05-2021).tif]

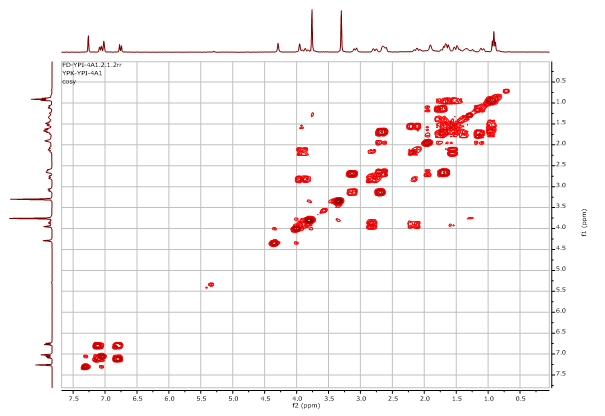

Supplement: Supplementary file 1 [file plants-10-01280-s001.zip › Figure S2 (14-05-2021).tif]

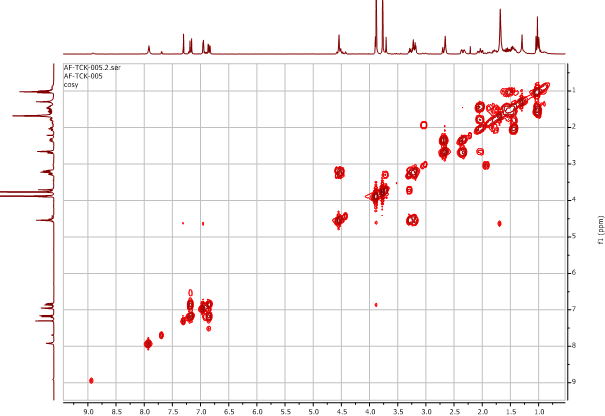

Supplement: Supplementary file 1 [file plants-10-01280-s001.zip › Figure S3 (14-05-2021).tif]

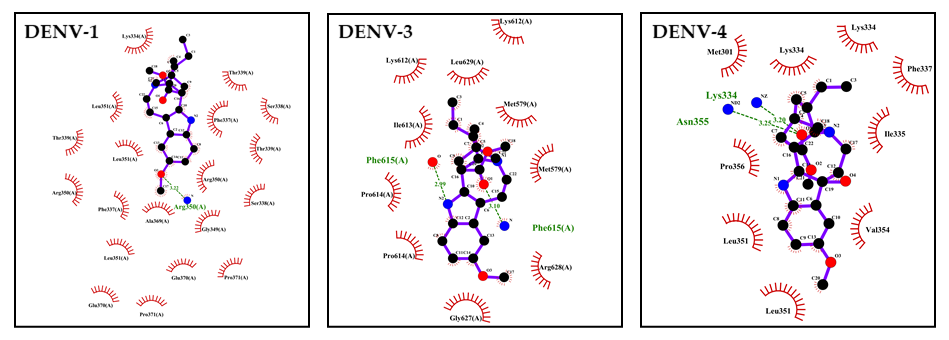

Supplement: Supplementary file 1 [file plants-10-01280-s001.zip › Figure S4 (14-05-2021).tif]
